# Percentile Curves for Anthropometric Measures for Canadian Children and Youth

**DOI:** 10.1371/journal.pone.0132891

**Published:** 2015-07-15

**Authors:** Stefan Kuhle, Bryan Maguire, Nicole Ata, David Hamilton

**Affiliations:** 1 Depts. of Pediatrics and Obstetrics & Gynaecology, Dalhousie University, Halifax, NS, Canada; 2 Dept. of Mathematics & Statistics, Dalhousie University, Halifax, NS, Canada; 3 School of Health and Human Performance, Dalhousie University, Halifax, NS, Canada; University of the Balearic Islands, SPAIN

## Abstract

Body mass index (BMI) is commonly used to assess a child's weight status but it does not provide information about the distribution of body fat. Since the disease risks associated with obesity are related to the amount and distribution of body fat, measures that assess visceral or subcutaneous fat, such as waist circumference (WC), waist-to-height ratio (WHtR), or skinfolds thickness may be more suitable. The objective of this study was to develop percentile curves for BMI, WC, WHtR, and sum of 5 skinfolds (SF5) in a representative sample of Canadian children and youth. The analysis used data from 4115 children and adolescents between 6 and 19 years of age that participated in the Canadian Health Measures Survey Cycles 1 (2007/2009) and 2 (2009/2011). BMI, WC, WHtR, and SF5 were measured using standardized procedures. Age- and sex-specific centiles were calculated using the LMS method and the percentiles that intersect the adult cutpoints for BMI, WC, and WHtR at age 18 years were determined. Percentile curves for all measures showed an upward shift compared to curves from the pre-obesity epidemic era. The adult cutoffs for overweight and obesity corresponded to the 72^nd^ and 91^st^ percentile, respectively, for both sexes. The current study has presented for the first time percentile curves for BMI, WC, WHtR, and SF5 in a representative sample of Canadian children and youth. The percentile curves presented are meant to be descriptive rather than prescriptive as associations with cardiovascular disease markers or outcomes were not assessed.

## Introduction

Childhood obesity is associated with adverse health, psychosocial, and economic outcomes in childhood and adulthood [1]. Body mass index (BMI) is the most commonly used method to assess a child's weight status. The drawbacks of BMI are that it cannot differentiate between lean and fat mass and does not provide information about the distribution of body fat [2–4]. Since the cardiovascular disease (CVD) risks associated with obesity are related to the amount and distribution of body fat [5–8], measures that assess visceral or subcutaneous fat may provide a better risk assessment than the BMI. Waist circumference (WC) and waist-to-height ratio (WHtR) have both been shown to be associated with CVD risk in children and adults [6,9–11]; skinfolds thickness (SF) can potentially better identify children and adults with excess total body fatness or adverse CVD risk factor levels than BMI [12–14]. As body composition in childhood is dependent on age, sex, and ethnicity, no single universal cut-off point exists for either of these measures in children and youth. Percentile curves have been developed for WC, WHtR, and SF in different populations [15–18] but, with the exception of waist circumference in youth aged 11 to 18 years [19], there are no Canadian reference data available. Therefore, the objective of the present paper was to develop percentile curves for anthropometric measures in a representative sample of Canadian children and youth using the LMS method [20].

## Materials and Methods

The current study used data from the Canadian Health Measures Survey (CHMS) Cycles 1 and 2. The CMHS is a representative, cross-sectional survey assessing indicators of health and wellness in Canadians between 3 and 79 years [21,22]. The survey consisted of a household interview to obtain sociodemographic and health information, and a visit to mobile examination centre to perform a number of physical measurements and tests. The sampling frame of the Canadian Labour Force Survey was used to identify the collection sites for the mobile examination centres. Within each collection site, households were selected using the 2006 Census as the sampling frame. Interviews and examinations for the CHMS Cycle 1 and 2 were performed between 2007 and 2009, and 2009 and 2011, respectively. The overall response rate in the two cycles was 51.7% and 55.7%, respectively. Data from the two cycles was combined as per Statistics Canada guidelines [23]. A total of 11,999 persons participated in physical examination part of the survey. The present analysis uses data from 4115 children and adolescents (2089 males and 2026 females) between 6 and 19 years of age.

### Anthropometric measures

All anthropometric measurements were performed by trained health professionals at the mobile examination centres. Body mass index was calculated from measured weight and height using the formula weight/height^2^ [kg/m^2^]. Weight was measured using a calibrated digital scale (Mettler Toledo, Mississauga, ON, Canada) to the nearest 0.1 kg. Standing height was measured using a fixed stadiometer with a vertical backboard and a moveable headboard to the nearest 0.01 cm. Waist circumference measurement was based on the Canadian Physical Activity, Fitness, and Lifestyle Approach (CPAFLA) protocol [24] using a 150 cm or a 200 cm Gulick tape measure. The WC was measured at the mid-point between the bottom of the rib cage and the top of the iliac crest at the end of a normal expiration to the nearest 0.1 cm. Waist to Height Ratio was calculated as waist circumference over height. Sum of 5 skinfolds (SF5) was determined using the five site method of the CPAFLA protocol [24] with a Harpenden skinfold caliper and a 150 cm Gulick tape measure to the nearest 0.2 mm. Each SF was measured twice. Triceps SF was measured on the midline of the back of the arm at the mid-point level between the acromium process and the tip of the olecranon process. Biceps SF was measured over the biceps at the same level as the midpoint for the triceps. Subscapular SF was measured below the inferior angle of the scapula at an angle of 45 degrees to the spine. Iliac crest SF was measured in the mid-axillary line above the crest of the ilium. Medial calf SF was measured at the medial side of the calf at the point of the largest circumference. Body mass index and WC were not measured in pregnant women, and SF measurements were not done on individuals with a BMI ≥ 30 kg/m^2^. Height was based on self-report for participants who were unable to stand unassisted.

### Statistical analysis

The data were stratified by sex and summarized using the LMS method by Cole and Green [20]. This method assumes that the data are normalized after the Box Cox transformation

$$z=\frac{{\left(y/\mu \right)}^{\lambda }-1}{\lambda \sigma }\;\;\lambda \neq 0$$ (1)

$$z=\frac{{\text{log}}_{e}(y/\mu )}{\sigma }\;\;\lambda =0$$ (2)

The age-specific distribution expresses the mean, coefficient of variation, and skewness as parameters that change smoothly as a function of age by modeling them as cubic splines. These functions can be plotted as smooth curves over age and are referred to as the M (mean μ), S (variance σ), and L (skewness λ) curves. Centiles are computed by using the values of the three parameters curves for a corresponding age with the formula

$$C_{100\alpha }=M{\left(1+L\times S\times z_{\alpha }\right)}^{1/L}$$ (3)

where z_α_ is the upper α quantile for the truncated standard normal distribution.

The LMS method allows us to assess the likelihood of an individual observation with the formula

$$z=\frac{{\left(y/M\right)}^{L}-1}{L\times S}$$ (4)

The 3^rd^, 10^th^, 25^th^, 50^th^, 75^th^, 90^th^, and 97^th^ centile curves were computed for BMI, WC, SF5, and WHtR. To avoid unusual behaviours of the spline functions near the end of the age range, data from respondents up to age 30 years was used to fit the models. This modification produced smoother curves that more accurately reflect the population characteristics. In addition, the z-score and percentile that intersects the adult cutpoints for BMI (18.5, 25, and 30 kg/m^2^) [25,26], WC (102 cm for males and 88 cm for females) [27], and WHtR (0.5 for both sexes) [28] at age 18 years were determined. Residual quantile plots ("worm plots") [29] were used to assess the goodness of fit of each component of the LMS models.

All calculations were performed using the sampling weights provided by Statistics Canada [23] to account for design effect and non-response bias. The CHMS uses a multistage sampling design with two sampling frames to select its sample. The probability of an individual to be selected for the survey is determined as the product of the probability of selection at each stage. To correct for non-response the weight of non-respondent households and individuals is redistributed to respondents within homogeneous response groups based on characteristics that are available for both respondents and non-respondents as determined from the Census of Canada (such as dwelling type or household income). A detailed description of the weighting procedure can be found elsewhere [22].

The statistical software package R [30] with the *gamlss* package [31] was used to perform the statistical analyses.

### Ethics statement

All processes used for cycles 1 and 2 of the CHMS were reviewed and approved by the Health Canada Research Ethics Board to ensure that internationally recognized ethical standards for human research were met and maintained. Written informed consent was obtained from all participants; parents or guardians gave consent on behalf of children aged 6 to 13 years, while the child provided his or her assent to participate [21,22]. The current project was approved by the IWK Health Centre Research Ethics Board, Halifax, NS, Canada (File # 1014413).

## Results

Descriptive statistics for BMI, WC, SF5, and WHtR by age and sex are shown in Table 1. The prevalence of overweight and obesity in the sample based on the IOTF (International Obesity Task Force) growth reference [32] was 17.0 and 9.6%, respectively. Characteristics of the sample are shown in S1 Table.

**Table 1:** Sample size, mean, and standard deviation for body mass index [kg/m^2^], waist circumference [cm], waist-to-height ratio, and sum of 5 skinfolds [mm] for Canadian children and youth aged 6 to 19 years.

			BMI		WC		WHtR		SF5	
Sex	Age [years]	n	Mean	SD	Mean	SD	Mean	SD	Mean	SD
Female	6	155	15.9	2.0	53.1	4.8	0.44	0.04	39.6	13.9
	7	140	16.3	2.4	54.6	6.6	0.44	0.05	44.1	19.6
	8	165	17.4	3.3	59.0	8.9	0.45	0.06	51.9	23.8
	9	177	18.1	3.3	61.1	9.0	0.45	0.06	60.0	29.7
	10	194	18.1	3.0	62.1	8.0	0.43	0.05	57.0	22.8
	11	216	19.4	3.6	65.7	10.4	0.44	0.06	60.4	25.3
	12	131	20.3	3.8	68.9	10.1	0.44	0.06	61.5	24.3
	13	132	20.5	3.5	69.8	9.0	0.43	0.05	68.9	30.5
	14	122	21.9	3.7	71.9	8.7	0.45	0.05	74.9	28
	15	129	23.7	5.5	77.0	14.1	0.47	0.08	77.1	31.6
	16	121	22.9	4.5	73.6	9.8	0.45	0.06	72.9	21.1
	17	125	23.3	4.7	75.8	13.0	0.46	0.08	77.2	26.1
	18	118	23.4	4.7	76.2	11.8	0.46	0.07	76.1	24.8
	19	101	23.4	4.2	75.9	9.3	0.46	0.06	78.5	23.4
Male	6	152	16.1	1.7	53.8	4.9	0.45	0.04	37.5	17.4
	7	165	17.9	3.7	59.3	10.1	0.47	0.07	47.6	28.7
	8	168	17.6	2.8	60.3	7.5	0.45	0.05	47.1	24.7
	9	166	18.7	3.5	63.4	9.9	0.46	0.06	52.2	28.1
	10	208	19.3	4.2	66.2	10.6	0.46	0.06	60.5	32.6
	11	193	19.1	3.7	66.1	9.7	0.45	0.06	53.2	29.9
	12	155	20.1	5.0	69.4	13.6	0.45	0.07	56.1	33.8
	13	145	20.5	4.0	71.9	11.3	0.44	0.06	55.0	30.8
	14	144	21.6	5.8	74.6	13.8	0.44	0.08	47.2	22.3
	15	130	22.4	4.8	76.1	12.6	0.44	0.07	44.7	21.9
	16	147	23.2	5.5	79.0	14.1	0.45	0.08	45.3	22.0
	17	123	23.6	4.7	80.4	11.6	0.45	0.07	46.5	19.0
	18	101	24.7	4.7	83.3	12.2	0.47	0.07	50.2	21.0
	19	92	25.1	5.2	83.7	12.1	0.47	0.07	53.4	20.5

Abbreviations: *BMI* Body Mass Index; *WC* Waist circumference; *WHtR* Waist-to-height ratio; *SF5* Sum of 5 skinfolds; *SD* Standard deviation.

**Fig 1:**
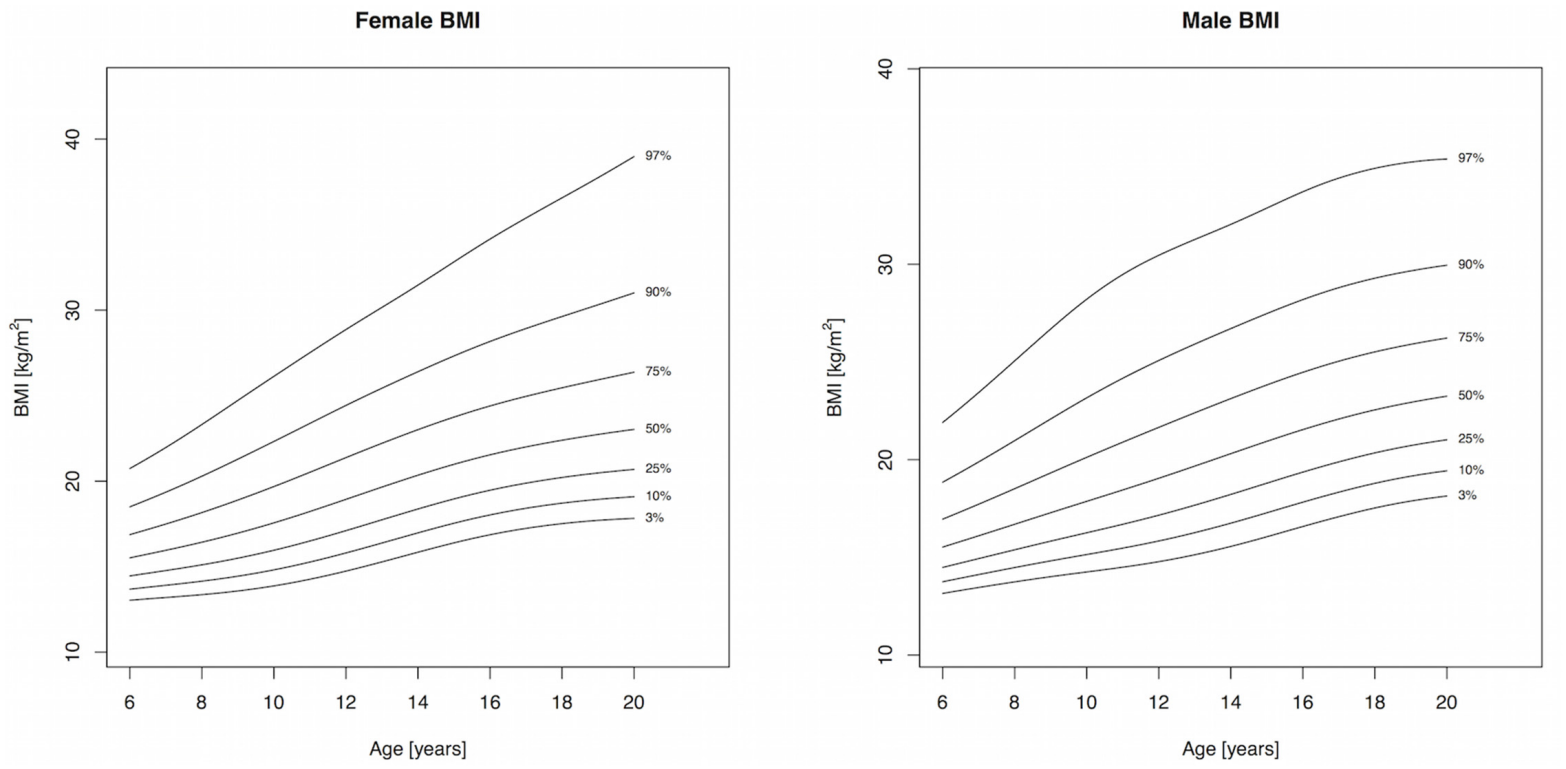
Percentile curves for body mass index for male and female Canadian children and youth aged 6 to 19 years.

**Fig 2:**
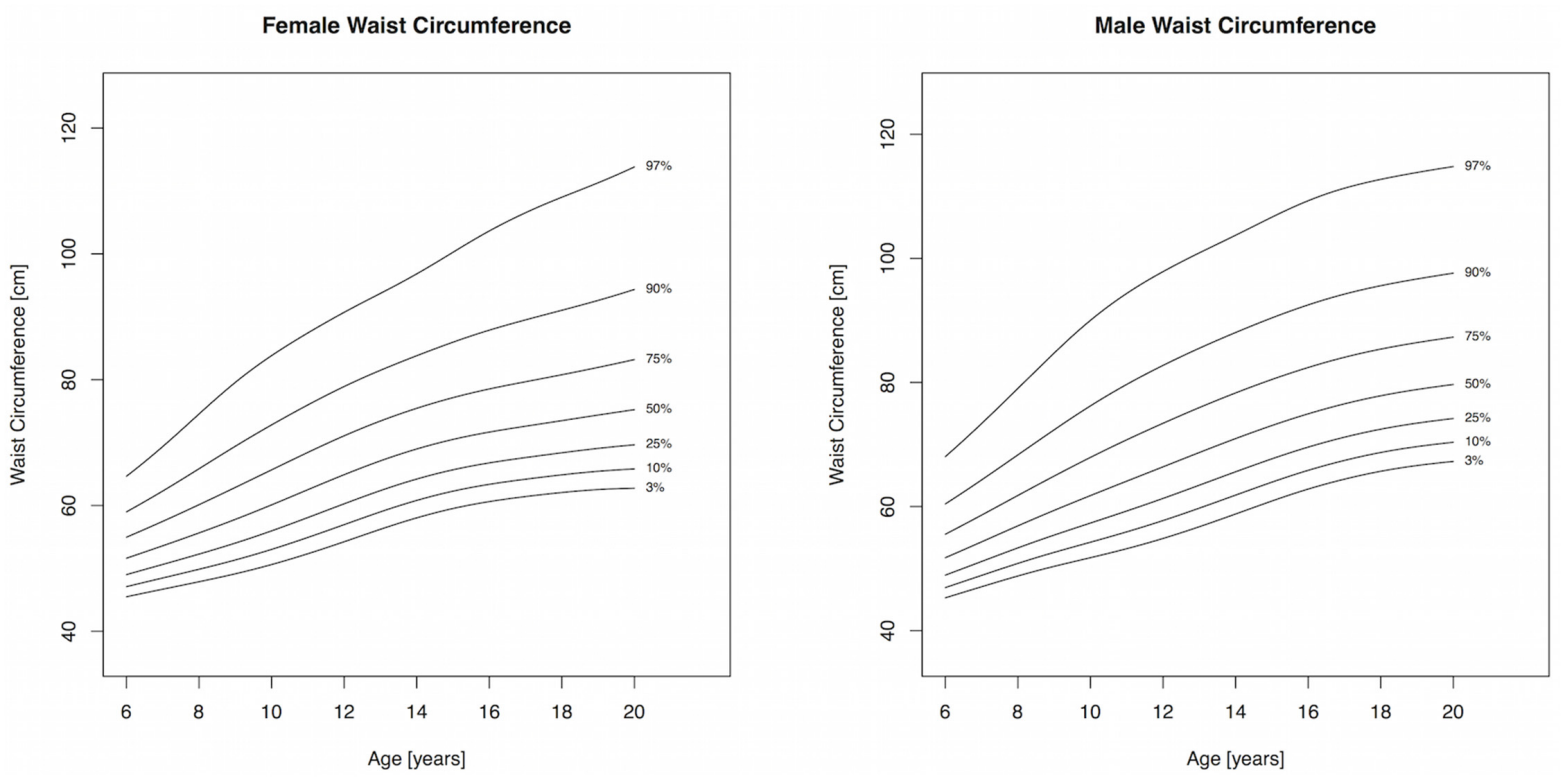
Percentile curves for waist circumference for male and female Canadian children and youth aged 6 to 19 years.

**Fig 3:**
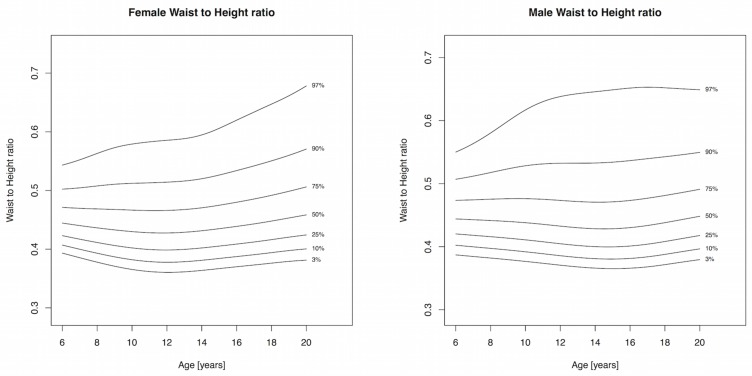
Percentile curves for waist to height ratio for male and female Canadian children and youth aged 6 to 19 years.

**Fig 4:**
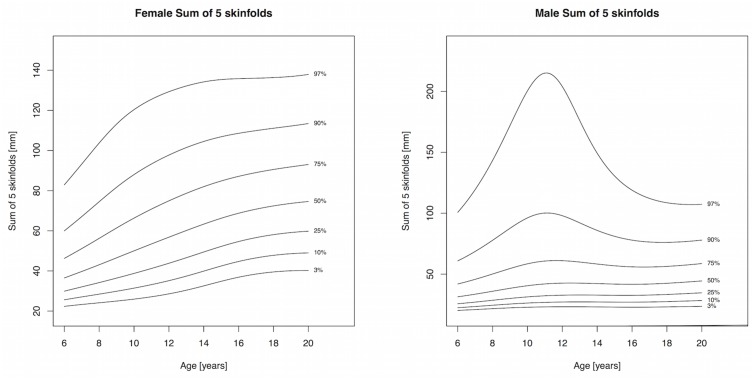
Percentile curves for sum of 5 skinfolds for male and female Canadian children and youth aged 6 to 19 years.

Body mass index and WC increased throughout childhood and percentile cutpoints were consistently higher in males compared to females, albeit the differences were small (Tables 2 and 3, Figs 1 and 2). The adult cutoffs for overweight and obesity approximately corresponded to the 72^nd^ and 91^st^ percentile, respectively, for both sexes. The 75^th^ percentile and lower for WHtR in girls showed a slight decline until 12 years, after which it increased, while the 90^th^ and 97^th^ percentile increased throughout childhood and adolescence; a similar pattern was observed for boys (Table 4 and Fig 3). The adult WHtR cutoff of 0.5 corresponded to the 81^st^ and 78^th^ percentile in males and females, respectively. Sum of 5 skinfolds increased until puberty in girls and plateaued afterwards. Among boys, the 50^th^ percentile for SF5 increased slightly until puberty and remained fairly constant afterwards, while percentile levels above the median exhibited a sharp increase with a peak around 11 years of age and subsequent drop (Table 5 and Fig 4). All three components of the models for females showed extremely good fit for every measure and a good fit for males with the exception of SF5, where the worm plots showed a moderate fit due to some kurtosis that the model could not account for.

**Table 2:** L, M, and S values, and percentiles of body mass index [kg/m^2^] by age and sex for Canadian children and youth aged 6 to 19 years.

Sex	Age [years]	L	M	S	3^rd^	10^th^	25^th^	50^th^	75^th^	90^th^	97^th^
Female	6	-2.2473	15.5148	0.1134	13.03	13.68	14.46	15.51	16.87	18.50	20.74
	6.5	-2.1695	15.7339	0.1186	13.12	13.80	14.62	15.73	17.18	18.92	21.34
	7	-2.0918	15.9561	0.1240	13.20	13.91	14.77	15.96	17.49	19.36	21.97
	7.5	-2.0148	16.1864	0.1296	13.28	14.03	14.93	16.19	17.82	19.81	22.63
	8	-1.9397	16.4293	0.1351	13.36	14.15	15.11	16.43	18.16	20.29	23.31
	8.5	-1.8678	16.6871	0.1405	13.46	14.29	15.29	16.69	18.52	20.78	24.01
	9	-1.8015	16.9617	0.1455	13.58	14.44	15.50	16.96	18.90	21.29	24.72
	9.5	-1.7419	17.2531	0.1500	13.72	14.62	15.72	17.25	19.28	21.80	25.43
	10	-1.6907	17.5612	0.1539	13.87	14.81	15.96	17.56	19.68	22.32	26.13
	10.5	-1.6492	17.8863	0.1572	14.06	15.03	16.22	17.89	20.10	22.85	26.82
	11	-1.6173	18.2266	0.1599	14.26	15.27	16.50	18.23	20.52	23.38	27.51
	11.5	-1.5959	18.5779	0.1620	14.49	15.53	16.80	18.58	20.95	23.91	28.20
	12	-1.5861	18.9357	0.1635	14.74	15.80	17.11	18.94	21.38	24.43	28.86
	12.5	-1.5876	19.2958	0.1645	15.00	16.09	17.42	19.30	21.80	24.94	29.52
	13	-1.6007	19.6537	0.1649	15.28	16.38	17.74	19.65	22.21	25.44	30.16
	13.5	-1.6265	20.0053	0.1650	15.56	16.68	18.06	20.00	22.62	25.92	30.81
	14	-1.6649	20.3473	0.1650	15.84	16.98	18.37	20.35	23.01	26.40	31.47
	14.5	-1.7134	20.6753	0.1648	16.12	17.26	18.67	20.67	23.39	26.87	32.14
	15	-1.7672	20.9855	0.1648	16.39	17.54	18.96	20.98	23.75	27.32	32.82
	15.5	-1.8208	21.2746	0.1648	16.64	17.79	19.23	21.27	24.08	27.76	33.50
	16	-1.8696	21.5409	0.1650	16.86	18.02	19.47	21.54	24.40	28.17	34.15
	16.5	-1.9106	21.7842	0.1654	17.06	18.23	19.69	21.78	24.69	28.56	34.78
	17	-1.9430	22.0064	0.1661	17.24	18.41	19.88	22.00	24.96	28.92	35.39
	17.5	-1.9666	22.2105	0.1671	17.39	18.57	20.06	22.21	25.21	29.28	35.98
	18	-1.9809	22.3996	0.1685	17.52	18.71	20.21	22.39	25.46	29.63	36.57
	18.5	-1.9861	22.5752	0.1701	17.62	18.83	20.35	22.57	25.69	29.97	37.15
	19	-1.9830	22.7388	0.1722	17.71	18.93	20.48	22.73	25.92	30.31	37.75
Male	6	-3.0597	15.5312	0.1150	13.16	13.75	14.48	15.53	16.96	18.85	21.90
	6.5	-2.9522	15.8204	0.1198	13.31	13.94	14.71	15.81	17.33	19.36	22.66
	7	-2.8446	16.1117	0.1248	13.46	14.12	14.94	16.11	17.72	19.89	23.44
	7.5	-2.7365	16.4056	0.1299	13.61	14.30	15.16	16.40	18.12	20.43	24.24
	8	-2.6273	16.7017	0.1351	13.75	14.48	15.39	16.69	18.52	20.98	25.06
	8.5	-2.5167	16.9981	0.1403	13.88	14.65	15.61	16.99	18.92	21.53	25.88
	9	-2.4055	17.2928	0.1455	14.01	14.82	15.83	17.29	19.32	22.08	26.69
	9.5	-2.2935	17.5845	0.1506	14.14	14.99	16.05	17.58	19.72	22.63	27.47
	10	-2.1809	17.8738	0.1555	14.25	15.15	16.26	17.87	20.12	23.16	28.21
	10.5	-2.0696	18.1628	0.1600	14.37	15.31	16.47	18.16	20.51	23.68	28.88
	11	-1.9615	18.4536	0.1640	14.50	15.47	16.69	18.45	20.90	24.17	29.48
	11.5	-1.8592	18.7487	0.1675	14.63	15.65	16.92	18.75	21.28	24.63	30.00
	12	-1.7656	19.0503	0.1702	14.78	15.84	17.16	19.05	21.65	25.08	30.46
	12.5	-1.6836	19.3586	0.1723	14.95	16.05	17.41	19.36	22.03	25.50	30.88
	13	-1.6157	19.6726	0.1737	15.13	16.27	17.67	19.67	22.40	25.91	31.27
	13.5	-1.5631	19.9897	0.1745	15.34	16.50	17.94	19.99	22.76	26.31	31.65
	14	-1.5283	20.3079	0.1747	15.56	16.75	18.22	20.31	23.12	26.71	32.04
	14.5	-1.5133	20.6252	0.1745	15.80	17.01	18.51	20.62	23.48	27.10	32.45
	15	-1.5157	20.9386	0.1738	16.06	17.28	18.80	20.94	23.82	27.48	32.87
	15.5	-1.5315	21.2443	0.1729	16.32	17.56	19.08	21.24	24.15	27.84	33.30
	16	-1.5546	21.5386	0.1717	16.58	17.83	19.36	21.54	24.47	28.19	33.71
	16.5	-1.5799	21.8184	0.1703	16.84	18.09	19.63	21.82	24.77	28.51	34.08
	17	-1.6043	22.0812	0.1689	17.08	18.34	19.89	22.08	25.04	28.80	34.41
	17.5	-1.6267	22.3250	0.1674	17.31	18.57	20.13	22.32	25.29	29.06	34.68
	18	-1.6444	22.5488	0.1659	17.53	18.79	20.35	22.55	25.52	29.29	34.91
	18.5	-1.6535	22.7515	0.1646	17.72	18.98	20.55	22.75	25.72	29.49	35.09
	19	-1.6519	22.9341	0.1636	17.88	19.16	20.72	22.93	25.90	29.66	35.23
	19.5	-1.6385	23.0988	0.1628	18.02	19.30	20.88	23.10	26.07	29.82	35.33

**Table 3:** L, M, and S values, and percentiles of waist circumference [cm] by age and sex for Canadian children and youth aged 6 to 19 years.

Sex	Age [years]	L	M	S	3^rd^	10^th^	25^th^	50^th^	75^th^	90^th^	97^th^
Female	6	-3.3915	51.6141	0.0838	45.5	47.1	49.0	51.6	55.0	59.0	64.6
	6.5	-3.3430	52.6017	0.0882	46.1	47.8	49.8	52.6	56.2	60.6	66.9
	7	-3.2914	53.5964	0.0928	46.7	48.5	50.6	53.6	57.5	62.3	69.4
	7.5	-3.2316	54.6083	0.0975	47.3	49.2	51.4	54.6	58.8	64.0	71.9
	8	-3.1579	55.6442	0.1022	47.9	49.9	52.3	55.6	60.1	65.8	74.5
	8.5	-3.0675	56.7060	0.1067	48.5	50.6	53.1	56.7	61.5	67.6	77.1
	9	-2.9629	57.8014	0.1108	49.1	51.3	54.0	57.8	62.9	69.4	79.6
	9.5	-2.8507	58.9362	0.1142	49.8	52.2	55.0	58.9	64.3	71.1	81.8
	10	-2.7428	60.1069	0.1168	50.6	53.0	56.0	60.1	65.7	72.8	83.8
	10.5	-2.6492	61.3069	0.1187	51.4	54.0	57.0	61.3	67.1	74.4	85.7
	11	-2.5761	62.5198	0.1199	52.3	54.9	58.1	62.5	68.4	76.0	87.4
	11.5	-2.5319	63.7243	0.1206	53.3	55.9	59.2	63.7	69.8	77.5	89.1
	12	-2.5216	64.9012	0.1207	54.2	57.0	60.3	64.9	71.1	78.9	90.7
	12.5	-2.5425	66.0347	0.1202	55.2	58.0	61.3	66.0	72.3	80.2	92.2
	13	-2.5913	67.1044	0.1194	56.2	59.0	62.4	67.1	73.4	81.5	93.7
	13.5	-2.6659	68.0931	0.1186	57.2	59.9	63.3	68.1	74.5	82.7	95.2
	14	-2.7635	68.9946	0.1179	58.0	60.8	64.2	69.0	75.4	83.8	96.8
	14.5	-2.8772	69.8066	0.1175	58.8	61.6	65.0	69.8	76.3	84.9	98.5
	15	-2.9977	70.5274	0.1175	59.5	62.3	65.7	70.5	77.1	86.0	100.3
	15.5	-3.1135	71.1599	0.1179	60.1	62.9	66.3	71.1	77.9	87.0	102.0
	16	-3.2154	71.7150	0.1184	60.6	63.4	66.8	71.7	78.5	87.9	103.6
	16.5	-3.2974	72.2120	0.1192	61.0	63.8	67.2	72.2	79.1	88.7	105.2
	17	-3.3559	72.6752	0.1200	61.4	64.2	67.6	72.6	79.7	89.5	106.5
	17.5	-3.3867	73.1266	0.1210	61.8	64.5	68.0	73.0	80.2	90.3	107.8
	18	-3.3842	73.5772	0.1221	62.1	64.9	68.4	73.5	80.8	91.0	109.0
	18.5	-3.3482	74.0258	0.1234	62.3	65.2	68.7	73.9	81.4	91.8	110.1
	19	-3.2813	74.4653	0.1251	62.5	65.4	69.1	74.4	82.0	92.6	111.3
Male	6	-3.7503	51.7546	0.0923	45.3	46.9	48.9	51.7	55.5	60.4	68.0
	6.5	-3.6725	53.0313	0.0959	46.2	47.9	50.0	53.0	57.1	62.3	70.7
	7	-3.5935	54.3204	0.0996	47.1	48.9	51.1	54.3	58.6	64.3	73.4
	7.5	-3.5104	55.6184	0.1035	47.9	49.9	52.2	55.6	60.2	66.3	76.2
	8	-3.4188	56.9091	0.1075	48.8	50.8	53.3	56.9	61.8	68.3	79.0
	8.5	-3.3145	58.1754	0.1116	49.6	51.7	54.4	58.1	63.4	70.4	81.9
	9	-3.1969	59.4078	0.1158	50.4	52.6	55.4	59.4	64.9	72.4	84.7
	9.5	-3.0695	60.6104	0.1199	51.1	53.4	56.4	60.6	66.4	74.3	87.5
	10	-2.9409	61.7915	0.1237	51.8	54.2	57.3	61.8	67.9	76.2	90.0
	10.5	-2.8230	62.9574	0.1268	52.5	55.1	58.3	62.9	69.4	78.0	92.3
	11	-2.7237	64.1126	0.1292	53.2	55.9	59.3	64.1	70.8	79.7	94.4
	11.5	-2.6472	65.2622	0.1308	54.0	56.8	60.3	65.2	72.1	81.3	96.2
	12	-2.5944	66.4153	0.1316	54.9	57.7	61.3	66.4	73.4	82.7	97.9
	12.5	-2.5650	67.5713	0.1318	55.8	58.7	62.4	67.6	74.7	84.2	99.4
	13	-2.5593	68.7228	0.1314	56.7	59.7	63.4	68.7	75.9	85.5	100.9
	13.5	-2.5777	69.8591	0.1306	57.8	60.8	64.5	69.8	77.2	86.8	102.3
	14	-2.6227	70.9692	0.1294	58.8	61.8	65.6	71.0	78.3	88.1	103.7
	14.5	-2.6936	72.0456	0.1279	59.8	62.9	66.7	72.0	79.4	89.3	105.2
	15	-2.7826	73.0804	0.1264	60.9	63.9	67.7	73.1	80.5	90.4	106.6
	15.5	-2.8750	74.0609	0.1249	61.9	64.9	68.7	74.0	81.5	91.5	108.0
	16	-2.9565	74.9781	0.1236	62.8	65.8	69.6	74.9	82.4	92.5	109.3
	16.5	-3.0201	75.8236	0.1225	63.7	66.7	70.4	75.8	83.3	93.4	110.4
	17	-3.0677	76.5926	0.1215	64.4	67.4	71.2	76.6	84.1	94.2	111.3
	17.5	-3.1045	77.2835	0.1205	65.1	68.1	71.9	77.2	84.8	95.0	112.1
	18	-3.1311	77.8966	0.1198	65.7	68.7	72.5	77.9	85.4	95.6	112.7
	18.5	-3.1447	78.4340	0.1192	66.2	69.2	73.0	78.4	85.9	96.2	113.3
	19	-3.1459	78.9050	0.1189	66.6	69.7	73.4	78.9	86.4	96.7	113.8
	19.5	-3.1359	79.3258	0.1188	67.0	70.0	73.8	79.3	86.9	97.2	114.3

**Table 4:** L, M, and S values, and percentiles of waist-to-height ratio by age and sex for Canadian children and youth aged 6 to 19 years.

Sex	Age [years]	L	M	S	3^rd^	10^th^	25^th^	50^th^	75^th^	90^th^	97^th^
Female	6	-3.1268	0.4445	0.0793	0.39	0.41	0.42	0.44	0.47	0.50	0.54
	6.5	-3.0890	0.4423	0.0831	0.39	0.40	0.42	0.44	0.47	0.50	0.55
	7	-3.0498	0.4402	0.0871	0.39	0.40	0.42	0.44	0.47	0.50	0.55
	7.5	-3.0066	0.4382	0.0912	0.38	0.40	0.41	0.44	0.47	0.51	0.56
	8	-2.9570	0.4363	0.0954	0.38	0.39	0.41	0.44	0.47	0.51	0.56
	8.5	-2.9005	0.4345	0.0994	0.37	0.39	0.41	0.43	0.47	0.51	0.57
	9	-2.8404	0.4328	0.1030	0.37	0.39	0.41	0.43	0.47	0.51	0.57
	9.5	-2.7793	0.4313	0.1062	0.37	0.38	0.40	0.43	0.47	0.51	0.58
	10	-2.7248	0.4299	0.1087	0.37	0.38	0.40	0.43	0.47	0.51	0.58
	10.5	-2.6817	0.4288	0.1108	0.36	0.38	0.40	0.43	0.47	0.51	0.58
	11	-2.6476	0.4280	0.1125	0.36	0.38	0.40	0.43	0.47	0.51	0.58
	11.5	-2.6226	0.4276	0.1137	0.36	0.38	0.40	0.43	0.47	0.51	0.58
	12	-2.6075	0.4276	0.1144	0.36	0.38	0.40	0.43	0.47	0.51	0.59
	12.5	-2.6030	0.4280	0.1147	0.36	0.38	0.40	0.43	0.47	0.51	0.59
	13	-2.6132	0.4288	0.1148	0.36	0.38	0.40	0.43	0.47	0.52	0.59
	13.5	-2.6397	0.4300	0.1148	0.36	0.38	0.40	0.43	0.47	0.52	0.59
	14	-2.6814	0.4314	0.1149	0.36	0.38	0.40	0.43	0.47	0.52	0.59
	14.5	-2.7347	0.4331	0.1153	0.37	0.38	0.40	0.43	0.47	0.52	0.60
	15	-2.7938	0.4349	0.1158	0.37	0.38	0.41	0.43	0.47	0.53	0.61
	15.5	-2.8502	0.4369	0.1165	0.37	0.39	0.41	0.44	0.48	0.53	0.61
	16	-2.8972	0.4389	0.1174	0.37	0.39	0.41	0.44	0.48	0.53	0.62
	16.5	-2.9315	0.4410	0.1184	0.37	0.39	0.41	0.44	0.48	0.54	0.63
	17	-2.9518	0.4432	0.1194	0.37	0.39	0.41	0.44	0.49	0.54	0.63
	17.5	-2.9586	0.4455	0.1205	0.37	0.39	0.41	0.45	0.49	0.55	0.64
	18	-2.9522	0.4481	0.1217	0.38	0.39	0.42	0.45	0.49	0.55	0.65
	18.5	-2.9344	0.4507	0.1229	0.38	0.40	0.42	0.45	0.50	0.56	0.65
	19	-2.9065	0.4533	0.1245	0.38	0.40	0.42	0.45	0.50	0.56	0.66
Male	6	-2.5598	0.4441	0.0876	0.39	0.40	0.42	0.44	0.47	0.51	0.55
	6.5	-2.7150	0.4434	0.0901	0.39	0.40	0.42	0.44	0.47	0.51	0.56
	7	-2.8691	0.4428	0.0926	0.38	0.40	0.42	0.44	0.47	0.51	0.56
	7.5	-3.0197	0.4422	0.0951	0.38	0.40	0.42	0.44	0.47	0.51	0.57
	8	-3.1652	0.4416	0.0977	0.38	0.40	0.42	0.44	0.48	0.52	0.58
	8.5	-3.3039	0.4409	0.1003	0.38	0.40	0.41	0.44	0.48	0.52	0.59
	9	-3.4350	0.4402	0.1029	0.38	0.39	0.41	0.44	0.48	0.52	0.60
	9.5	-3.5583	0.4393	0.1054	0.38	0.39	0.41	0.44	0.48	0.53	0.61
	10	-3.6735	0.4384	0.1079	0.38	0.39	0.41	0.44	0.48	0.53	0.62
	10.5	-3.7799	0.4373	0.1102	0.38	0.39	0.41	0.44	0.48	0.53	0.62
	11	-3.8765	0.4362	0.1123	0.37	0.39	0.41	0.44	0.48	0.53	0.63
	11.5	-3.9622	0.4349	0.1142	0.37	0.39	0.41	0.43	0.47	0.53	0.63
	12	-4.0348	0.4337	0.1160	0.37	0.39	0.40	0.43	0.47	0.53	0.64
	12.5	-4.0930	0.4325	0.1176	0.37	0.38	0.40	0.43	0.47	0.53	0.64
	13	-4.1341	0.4314	0.1191	0.37	0.38	0.40	0.43	0.47	0.53	0.64
	13.5	-4.1552	0.4306	0.1203	0.37	0.38	0.40	0.43	0.47	0.53	0.64
	14	-4.1548	0.4301	0.1214	0.37	0.38	0.40	0.43	0.47	0.53	0.65
	14.5	-4.1327	0.4299	0.1222	0.37	0.38	0.40	0.43	0.47	0.53	0.65
	15	-4.0898	0.4300	0.1228	0.37	0.38	0.40	0.43	0.47	0.53	0.65
	15.5	-4.0283	0.4306	0.1231	0.37	0.38	0.40	0.43	0.47	0.54	0.65
	16	-3.9500	0.4316	0.1232	0.37	0.38	0.40	0.43	0.47	0.54	0.65
	16.5	-3.8599	0.4329	0.1230	0.37	0.38	0.40	0.43	0.47	0.54	0.65
	17	-3.7677	0.4346	0.1225	0.37	0.38	0.40	0.43	0.48	0.54	0.65
	17.5	-3.6821	0.4366	0.1218	0.37	0.39	0.41	0.44	0.48	0.54	0.65
	18	-3.6020	0.4388	0.1209	0.37	0.39	0.41	0.44	0.48	0.54	0.65
	18.5	-3.5230	0.4412	0.1200	0.37	0.39	0.41	0.44	0.48	0.54	0.65
	19	-3.4418	0.4436	0.1192	0.38	0.39	0.41	0.44	0.49	0.55	0.65
	19.5	-3.3555	0.4461	0.1185	0.38	0.39	0.42	0.45	0.49	0.55	0.65

**Table 5:** L, M, and S values, and percentiles of sum of 5 skinfolds [mm] by age and sex for Canadian children and youth aged 6 to 19 years.

Sex	Age [years]	L	M	S	3^rd^	10^th^	25^th^	50^th^	75^th^	90^th^	97^th^
Female	6	-0.8001	36.4877	0.3200	22.3	25.6	29.9	36.5	46.2	60.0	82.9
	6.5	-0.7432	38.0983	0.3315	22.8	26.3	31.0	38.1	48.7	63.5	88.0
	7	-0.6868	39.7213	0.3433	23.3	27.0	32.0	39.7	51.1	67.1	93.2
	7.5	-0.6321	41.3740	0.3551	23.7	27.7	33.1	41.4	53.6	70.8	98.5
	8	-0.5792	43.0677	0.3663	24.1	28.4	34.2	43.1	56.2	74.5	103.7
	8.5	-0.5273	44.7916	0.3764	24.5	29.1	35.3	44.8	58.8	78.2	108.7
	9	-0.4769	46.5333	0.3851	25.0	29.9	36.4	46.5	61.4	81.7	113.2
	9.5	-0.4282	48.2768	0.3920	25.4	30.6	37.6	48.3	63.9	85.0	117.1
	10	-0.3824	50.0088	0.3967	25.9	31.4	38.8	50.0	66.3	88.0	120.4
	10.5	-0.3403	51.7288	0.3994	26.5	32.3	40.0	51.7	68.6	90.8	123.2
	11	-0.3019	53.4382	0.4002	27.1	33.2	41.2	53.4	70.8	93.3	125.5
	11.5	-0.2677	55.1324	0.3995	27.8	34.1	42.5	55.1	72.9	95.6	127.6
	12	-0.2384	56.8117	0.3971	28.6	35.1	43.8	56.8	74.9	97.7	129.3
	12.5	-0.2142	58.4805	0.3933	29.4	36.2	45.2	58.5	76.8	99.7	130.8
	13	-0.1952	60.1312	0.3881	30.4	37.4	46.6	60.1	78.7	101.5	132.2
	13.5	-0.1811	61.7488	0.3819	31.4	38.6	48.0	61.7	80.4	103.1	133.3
	14	-0.1711	63.3203	0.3748	32.5	39.9	49.4	63.3	82.0	104.5	134.2
	14.5	-0.1632	64.8268	0.3672	33.7	41.2	50.9	64.8	83.5	105.8	134.9
	15	-0.1556	66.2454	0.3596	34.8	42.5	52.2	66.2	84.8	106.9	135.4
	15.5	-0.1468	67.5584	0.3523	35.9	43.6	53.5	67.6	86.0	107.8	135.7
	16	-0.1357	68.7533	0.3458	36.9	44.7	54.6	68.8	87.1	108.6	135.8
	16.5	-0.1223	69.8262	0.3401	37.7	45.7	55.7	69.8	88.1	109.3	135.9
	17	-0.1066	70.7856	0.3355	38.4	46.5	56.6	70.8	89.0	109.9	136.0
	17.5	-0.0893	71.6414	0.3319	39.0	47.2	57.4	71.6	89.8	110.5	136.2
	18	-0.0704	72.3989	0.3292	39.5	47.8	58.1	72.4	90.6	111.1	136.4
	18.5	-0.0505	73.0634	0.3275	39.8	48.2	58.7	73.1	91.2	111.7	136.6
	19	-0.0302	73.6442	0.3266	40.1	48.6	59.1	73.6	91.9	112.2	136.9
Male	6	-2.3230	38.7422	0.7465	20.3	22.5	25.7	31.4	41.9	60.9	100.7
	6.5	-2.2059	39.3403	0.7137	20.6	23.0	26.4	32.5	43.8	64.6	109.5
	7	-2.0893	39.9486	0.6823	21.0	23.4	27.1	33.6	45.8	68.6	119.4
	7.5	-1.9737	40.5630	0.6521	21.3	24.0	27.8	34.7	47.9	73.1	130.7
	8	-1.8589	41.1632	0.6236	21.7	24.5	28.6	36.0	50.1	77.8	143.3
	8.5	-1.7449	41.7239	0.5969	22.1	25.0	29.3	37.2	52.4	82.7	157.2
	9	-1.6319	42.2178	0.5723	22.4	25.5	30.1	38.4	54.6	87.7	172.0
	9.5	-1.5210	42.6277	0.5499	22.6	25.9	30.8	39.5	56.7	92.3	186.9
	10	-1.4144	42.9405	0.5294	22.9	26.3	31.4	40.5	58.5	96.2	200.4
	10.5	-1.3138	43.1455	0.5108	23.0	26.6	31.9	41.4	59.9	98.9	210.5
	11	-1.2209	43.2441	0.4938	23.1	26.8	32.3	42.0	60.8	100.1	215.0
	11.5	-1.1370	43.2479	0.4782	23.2	27.0	32.6	42.4	61.1	99.8	212.7
	12	-1.0626	43.1711	0.4639	23.2	27.1	32.8	42.6	61.1	98.0	204.0
	12.5	-0.9971	43.0229	0.4510	23.2	27.2	32.9	42.7	60.6	95.3	190.8
	13	-0.9398	42.8208	0.4393	23.2	27.2	32.9	42.6	59.9	92.1	176.0
	13.5	-0.8895	42.5825	0.4289	23.2	27.2	32.9	42.5	59.1	88.9	161.8
	14	-0.8448	42.3307	0.4197	23.1	27.2	32.8	42.3	58.3	85.9	149.2
	14.5	-0.8044	42.0880	0.4118	23.0	27.1	32.7	42.1	57.5	83.3	138.9
	15	-0.7672	41.8827	0.4053	23.0	27.0	32.7	41.9	56.9	81.1	130.5
	15.5	-0.7323	41.7453	0.3999	22.9	27.0	32.6	41.7	56.4	79.3	124.0
	16	-0.6987	41.6987	0.3955	22.9	27.0	32.6	41.7	56.0	77.9	118.9
	16.5	-0.6656	41.7543	0.3922	22.9	27.1	32.7	41.8	55.9	77.0	115.1
	17	-0.6326	41.9139	0.3897	23.0	27.2	32.9	41.9	55.9	76.4	112.2
	17.5	-0.6000	42.1743	0.3878	23.0	27.3	33.1	42.2	56.1	76.1	110.1
	18	-0.5676	42.5227	0.3866	23.1	27.5	33.3	42.5	56.4	76.1	108.6
	18.5	-0.5358	42.9398	0.3860	23.3	27.7	33.7	42.9	56.8	76.3	107.7
	19	-0.5045	43.4095	0.3860	23.4	27.9	34.0	43.4	57.4	76.7	107.2
	19.5	-0.4735	43.9177	0.3867	23.5	28.1	34.3	43.9	58.0	77.3	107.1

Details on the percentiles of BMI, WC, and WHtR by age and sex that intersect adult cutpoints at age 18 years can be found in Table 6 and S2 Table.

**Table 6:** Z-scores and percentiles that intersect the adult cutpoints for body mass index, waist circumference, and waist-to-height ratio at age 18 years.

	Adult cutpoint	z-score	Percentile
**Body mass index—Thinness Grade 1**			
Male	18.5 kg/m^2^	-1.41	7.9
Female	18.5 kg/m^2^	-1.38	8.4
**Body mass index—Overweight**			
Male	25 kg/m^2^	0.57	71.6
Female	25 kg/m^2^	0.59	72.1
**Body mass index—Obesity**			
Male	30 kg/m^2^	1.37	91.5
Female	30 kg/m^2^	1.32	90.6
**Waist circumference**			
Male	102 cm	1.52	93.6
Female	88 cm	1.10	86.4
**Waist-to-Height ratio**			
Male	0.5	0.86	80.6
Female	0.5	0.77	77.9

## Discussion

The current study has presented for the first time percentile curves for the most commonly used anthropometric measures for body composition assessment based on a representative sample of Canadian children and youth aged 6 to 19 years. The percentile curves presented are meant to be descriptive rather than prescriptive as associations with cardiovascular disease markers or outcomes were not assessed. The data may be used by researchers as reference data for future studies.

The general shape of the BMI centile curves in males and females was comparable to existing growth curves from the IOTF [32], WHO (World Health Organization) [33], and CDC (Centers for Disease Control) [34] with the BMI increasing steadily until puberty, after which the slope begins to level off. Since the data for the CHMS were collected during the obesity epidemic, the percentile cutpoints are higher than those from the IOTF, WHO, and CDC curves, which were based on data that were for the most part collected before the 1980s. While the 72^nd^ and 91^st^ percentiles for both sexes in our sample intersected the adult cutpoints for overweight and obesity at age 18 years, the corresponding percentiles were 88^th^ (females) and 90^th^ (males) for overweight, and 99^th^ for obesity (both sexes) using the IOTF reference [32], and 82^nd^ (females) and 81^th^ (males) for overweight, and 95^th^ (females) and 96^th^ (males) for obesity using the CDC reference [34]. These differences underline the importance of relating percentile curves and cutoffs to actual health outcomes as the associated health risks of distribution-based cutoffs may change considerably with the reference population.

Waist circumference has been found to be a better predictor of CVD risk factors in children than BMI [6,10,35] but the method still has not seen widespread use, neither clinically or for research purposes. Percentiles for WC in children have been published for more than 20 countries but there is no universally accepted WC cutoff that is based on health outcomes or CVD risk markers in children. Establishment of WC cutoffs is further hampered by the use of different method to measure WC between studies. While the CHMS and other recent studies consistently measured the WC at the midpoint between the lower end of the rib cage and the iliac crest at the end of an expiration [16,18], studies in samples from the pre-obesity epidemic era often used the umbilicus or the point of maximum waist narrowing [17,19,36]. For this reason, comparison of WC percentiles determined in this study with existing Canadian reference data [19] from the 1981 Canadian Fitness Survey (which measured WC at *"the point of noticeable waist narrowing"*) was not possible. The shape of the WC curves in boys and girls in the present study is similar to previously published centile curves [16], including the Canadian reference curves [19]. The intersection of the WC curves at age 18 years with recommended cutoff points for adults suggested the 94^th^ percentile (males) and 86^th^ percentile (females), respectively, as WC cutoffs in childhood. The only other study that related their centile curves to adult cutoffs was a German study using data collected between 2003 and 2006; the investigators found that the 98^th^ and 97^th^ percentile corresponded to the adult cutoffs in males and females, respectively [16].

A WHtR greater than 0.5 has been proposed as an indicator of CVD risk in adults [28] and two recent meta-analyses concluded that WHtR provides a better discrimination for CVD outcomes in adults than WC or BMI [9,11]. The same cutoff has been suggested for use throughout childhood [37,38], which would offer the great advantage of obviating the need for age-related reference values and providing the simple universal public health message *"keep your waist circumference to less than half your height"* [38]. Centiles for WHtR in the present study, with the exception of the higher percentiles, decreased slightly until around puberty and increased again thereafter as reported previously in samples from other countries [18,39]. The median WHtR varied by less than 3% throughout childhood for both sexes. The percentiles that corresponded to the 0.5 cutoff at age 18 years were comparable between males and females (81^st^ and 78^th^ percentile, respectively). These cutoffs were lower than those found in representative samples of Norwegian [18] and German children and youth [39], where the cutoffs fell above the 90^th^ percentile.

Compared to the other methods of body fat measurement discussed above, skinfold thickness has the highest measurement error [40], which may further increase with the degree of adiposity [41]. This shortcoming makes interpretation of skinfolds thickness challenging and, along with the need for specialized measurement equipment (calipers), may account for its limited use. In the largest study to date, Addo and Himes determined reference curves for triceps and subscapular skinfold thickness based on five large representative samples of US children between 1963 and 1994 [15]. They found a distinct difference in skinfold trajectories between sexes, with boys exhibiting a dramatic increase in the percentile levels above the median before puberty while girls showed a more steady increase throughout childhood and adolescence. Sex differences were also evident for SF5 in the current study: Percentile levels above the 75^th^ percentile showed a steep increase before puberty for boys followed by a sharp decline as result of an increase and decline in the μ parameter that is amplified by a large variance and large negative value of the skewness curve in this age range. The percentiles for girls increased slightly until age 12 years and then remained fairly constant. Almost identical sex-specific patterns for triceps and subscapular skinfold thickness were found in a large representative sample of German children (n = 17,158) [42].

The strengths of the current study include the nationally representative sample of children and youth, and the use of sample weighting to account for non-response and design effect. Due to the physical burden of the assessments used in the survey, and the need to travel to the mobile examination clinics, there may have been a self-selection toward more mobile, healthier and fitter individuals. Our study is limited by the relatively small sample size, and the cross-sectional nature of the data; longitudinal data may more accurately reflect how body fatness changes with age. Lastly, while the LMS method provides a very flexible and powerful tool to model data whose dispersion and skewness change over age, it lacks a mechanism for modeling kurtosis, an issue addressed by extensions of the LMS method [43,44]. Its flexibility also means that the curves may differ considerably based on the parameter choices made by the researcher; e.g., the choice of a higher smoothing parameter allows the elimination of features that are not representative of the underlying population. While choice of smoothing parameters and model selection adds some arbitrariness to the process it allows the researcher to balance mathematical rigidity with clinical usefulness.

## Conclusions

This study has presented percentile curves for measures of body fatness in a representative sample of Canadian children and youth. Our findings indicate a substantial upward shift of the percentile curves for all measures compared to data from the pre-obesity epidemic era. Since we did not examine any relationships with health outcomes or disease markers, the data should be considered as a reference for future studies not as a growth standard.

## Supporting Information

S1 Table Characteristics of 4115 Canadian children and youth aged 6 to 19 years in the Canadian Health Measures Survey Cycles 1 and 2. (DOCX)

S2 Table Percentiles of body mass index, waist circumference, and waist-to-height ratio that intersect adult cutpoints at age 18 years. (DOCX)
