# Supplementary material for: Percentile Curves for Anthropometric Measures for Canadian Children and Youth
Source: PLoS One. 2015 Jul 15;10(7):e0132891. doi: 10.1371/journal.pone.0132891 (PMC4503699; doi:10.1371/journal.pone.0132891)
Supplement: S1 Table — (DOCX) [file pone.0132891.s001.docx]

**S1 Table: Characteristics of 4115 Canadian children and youth aged 6 to 19 years in the Canadian Health Measures Survey Cycles 1 and 2.**

|  | **Prevalence** [%] |
| --- | --- |
| **Sex** |  |
| Male | 51.5 |
| Female | 48.5 |
| **Region of Canada** |  |
| Atlantic Canada | 6.7^E^ |
| Québec | 22.5 |
| Ontario | 40.9 |
| Prairies | 17.8 |
| British Columbia | 12.1 |
| **Racial origin** |  |
| White | 83.3 |
| Black | 6.3^E^ |
| Asian | 8.1 |
| Other | 2.3^E^ |
| **Weight status** |  |
| Underweight | 7.2 |
| Normal weight | 66.2 |
| Overweight | 17.0 |
| Obese | 9.6 |
| **Household education** |  |
| Secondary school or less | 14.1 |
| College | 50.2 |
| University | 35.7 |
| **Household income** |  |
| $30,000 or less | 13.6 |
| $30,001 - $60,000 | 23.3 |
| $60,001 - $80,000 | 19.4 |
| $80,001 - $100,000 | 16.6 |
| > $100,000 | 27.1 |

^E^ Coefficient of variation between 16.6% and 33.3%; interpret with caution as per Statistics Canada sampling variability reporting guidelines.
