# Supplementary material for: Percentile Curves for Anthropometric Measures for Canadian Children and Youth
Source: PLoS One. 2015 Jul 15;10(7):e0132891. doi: 10.1371/journal.pone.0132891 (PMC4503699; doi:10.1371/journal.pone.0132891)
Supplement: S2 Table — (DOCX) [file pone.0132891.s002.docx]

**S2 Table:** **Percentiles of body mass index, waist circumference, and waist-to-height ratio that intersect adult cutpoints* at age 18 years.**

| **Sex** | **Age [years]** | **BMI Thinness** | **BMI Overweight** | **BMI Obesity** | **WC** | **WHtR** |
| --- | --- | --- | --- | --- | --- | --- |
| Female | 6 | 13.57 | 16.67 | 18.61 | 57.6 | 0.48 |
|  | 6.5 | 13.68 | 16.96 | 19.04 | 59.1 | 0.47 |
|  | 7 | 13.78 | 17.26 | 19.48 | 60.7 | 0.47 |
|  | 7.5 | 13.89 | 17.58 | 19.95 | 62.3 | 0.47 |
|  | 8 | 14.01 | 17.90 | 20.44 | 63.9 | 0.47 |
|  | 8.5 | 14.14 | 18.25 | 20.94 | 65.6 | 0.47 |
|  | 9 | 14.29 | 18.61 | 21.46 | 67.2 | 0.47 |
|  | 9.5 | 14.46 | 18.98 | 21.98 | 68.8 | 0.47 |
|  | 10 | 14.65 | 19.37 | 22.51 | 70.4 | 0.47 |
|  | 10.5 | 14.86 | 19.77 | 23.04 | 72.0 | 0.47 |
|  | 11 | 15.09 | 20.18 | 23.58 | 73.5 | 0.47 |
|  | 11.5 | 15.34 | 20.59 | 24.11 | 74.9 | 0.47 |
|  | 12 | 15.61 | 21.01 | 24.64 | 76.3 | 0.47 |
|  | 12.5 | 15.90 | 21.42 | 25.16 | 77.6 | 0.47 |
|  | 13 | 16.19 | 21.83 | 25.66 | 78.8 | 0.47 |
|  | 13.5 | 16.48 | 22.22 | 26.16 | 79.9 | 0.48 |
|  | 14 | 16.77 | 22.61 | 26.64 | 81.0 | 0.48 |
|  | 14.5 | 17.06 | 22.98 | 27.12 | 82.1 | 0.48 |
|  | 15 | 17.33 | 23.33 | 27.59 | 83.1 | 0.48 |
|  | 15.5 | 17.59 | 23.66 | 28.04 | 84.0 | 0.48 |
|  | 16 | 17.82 | 23.96 | 28.47 | 84.9 | 0.49 |
|  | 16.5 | 18.02 | 24.25 | 28.87 | 85.7 | 0.49 |
|  | 17 | 18.20 | 24.51 | 29.26 | 86.5 | 0.49 |
|  | 17.5 | 18.36 | 24.76 | 29.63 | 87.3 | 0.50 |
| Male | 6 | 13.62 | 16.72 | 19.27 | 63.2 | 0.48 |
|  | 6.5 | 13.79 | 17.08 | 19.81 | 65.3 | 0.48 |
|  | 7 | 13.97 | 17.45 | 20.38 | 67.6 | 0.48 |
|  | 7.5 | 14.14 | 17.83 | 20.95 | 69.9 | 0.49 |
|  | 8 | 14.31 | 18.21 | 21.54 | 72.3 | 0.49 |
|  | 8.5 | 14.48 | 18.60 | 22.12 | 74.6 | 0.49 |
|  | 9 | 14.64 | 18.98 | 22.70 | 77.0 | 0.49 |
|  | 9.5 | 14.79 | 19.36 | 23.28 | 79.2 | 0.49 |
|  | 10 | 14.94 | 19.74 | 23.83 | 81.3 | 0.49 |
|  | 10.5 | 15.09 | 20.11 | 24.35 | 83.2 | 0.49 |
|  | 11 | 15.25 | 20.47 | 24.84 | 84.9 | 0.49 |
|  | 11.5 | 15.42 | 20.84 | 25.31 | 86.6 | 0.49 |
|  | 12 | 15.60 | 21.20 | 25.75 | 88.1 | 0.49 |
|  | 12.5 | 15.79 | 21.57 | 26.17 | 89.5 | 0.49 |
|  | 13 | 16.01 | 21.93 | 26.59 | 90.9 | 0.49 |
|  | 13.5 | 16.23 | 22.29 | 26.99 | 92.3 | 0.49 |
|  | 14 | 16.48 | 22.64 | 27.38 | 93.6 | 0.49 |
|  | 14.5 | 16.73 | 22.99 | 27.77 | 94.9 | 0.49 |
|  | 15 | 17.00 | 23.33 | 28.16 | 96.2 | 0.49 |
|  | 15.5 | 17.27 | 23.65 | 28.53 | 97.5 | 0.49 |
|  | 16 | 17.54 | 23.97 | 28.89 | 98.7 | 0.50 |
|  | 16.5 | 17.80 | 24.26 | 29.22 | 99.7 | 0.50 |
|  | 17 | 18.05 | 24.53 | 29.51 | 100.6 | 0.50 |
|  | 17.5 | 18.28 | 24.78 | 29.77 | 101.3 | 0.50 |

* BMI thinness - 18.5 kg/m^2^ for both sexes; BMI overweight - 25.0 kg/m^2^ for both sexes; BMI obesity - 30.0 kg/m^2^ for both sexes; WC - 102 cm for males and 88 cm for females; WHtR - 0.5 for both sexes

Abbreviations: *BMI* Body Mass Index; *WC* Waist Circumference; *WHtR* Waist-To-Height Ratio
